# HIV Self-Testing among Men Who Have Sex with Men (MSM) in the UK: A Qualitative Study of Barriers and Facilitators, Intervention Preferences and Perceived Impacts

**DOI:** 10.1371/journal.pone.0162713

**Published:** 2016-09-09

**Authors:** T. Charles Witzel, Alison J. Rodger, Fiona M. Burns, Tim Rhodes, Peter Weatherburn

**Affiliations:** 1 Sigma Research, Department of Social and Environmental Health Research, Public Health and Policy, London School of Hygiene and Tropical Medicine, London, United Kingdom; 2 Research Department of Infection & Population Health, University College London, London, United Kingdom; 3 Department of Social and Environmental Health Research, Public Health and Policy, London School of Hygiene and Tropical Medicine, London, United Kingdom; National and Kapodistrian University of Athens, GREECE

## Abstract

**Introduction:**

Innovative strategies, such as HIV self-testing (HIVST), could increase HIV testing rates and diagnosis. Evidence to inform the design of an HIVST intervention in the UK is scarce with very little European data on this topic. This study aims to understand values and preferences for HIVST interventions targeting MSM in the UK. We explore the acceptability of HIVST among MSM in the context of known barriers and facilitators to testing for HIV; assess preferences for, and the concerns about, HIVST.

**Methods:**

Six focus group discussions (FGD) were conducted with 47 MSM in London, Manchester and Plymouth. HIVST as a concept was discussed and participants were asked to construct their ideal HIVST intervention. OraQuick^TM^ and BioSure^TM^ kits were then demonstrated and participants commented on procedure, design and instructions. FGDs were recorded and transcribed verbatim, then analysed thematically.

**Results:**

Convenience and confidentiality of HIVST was seen to facilitate testing. Issues with domestic privacy problematised confidentiality. HIVST kits and instructions were thought to be unnecessarily complicated, and did not cater to the required range of abilities. The window period was the most important element of an HIVST, with strong preference for 4^th^ generation testing. Kits which used a blood sample were more popular than those using saliva due to higher perceived accuracy although phobia of needles and/or blood meant some would only access HIVST if a saliva sample option was available. A range of access options was important to maintain convenience and privacy. HIVST kits were assumed to increase frequency of testing, with concerns related to the dislocation of HIVST from sexual health care pathways and services.

**Discussion:**

Utility of HIVST arises from relatively high levels of confidentiality and convenience. Until 4^th^ generation assays are available HIVST will be seen as supplementary in a UK context.

## Introduction

Reducing late HIV diagnosis is a UK public health priority which has led to the expansion of HIV testing outside clinical settings [1, 2]. Correspondingly, the volume of tests undertaken in the UK has increased dramatically, and the number of men who have sex with men (MSM) who have undiagnosed HIV has seen a steady decline over the past decade [1, 3].

HIV testing uptake and frequency remains sub-optimal however, with recent community surveys suggesting that approximately 25% of MSM have never tested for HIV and between 50–60% have not tested in the previous year [2, 4, 5]. An estimated 40% of MSM diagnosed with HIV in the UK are diagnosed late (defined as CD4 counts less than 350mm^3^), increasing the risk of HIV related morbidity and mortality [3].

Factors mediating MSM’s testing decision making are complex. Significant barriers impeding access to testing include fears of the implications of receiving a positive result, stigma, and structural and health service factors [6,7]. In an effort to address these, policy makers, health promoters and commissioners have made significant attempts to promote testing and have expanded the volume and variety of HIV testing services across the UK [1].

While the majority of HIV tests in the UK are conducted in genito-urinary medicine (GUM) clinics, the last 10 years has seen a substantial increase in other testing options [1]. Point of care (PoCT) (or rapid) testing (undertaken by another person such as a healthcare worker) is commonly offered in a wide variety of settings by community based organisations. England has a national HIV self-sampling (HIVSS) service, where an individual takes a sample which they then post back to the laboratory where it is processed and the patient is contacted with the result [8, 9].

Another approach is to offer HIV self-testing (HIVST) where the person takes a sample, conducts the test and reads the result themselves. Self-testing was legalised in the UK in April 2014, with the first kite-marked HIV self-testing kit released to the UK market in April 2015. This kit uses a whole blood sample and is marketed under the name BioSure^TM^. HIVST has the potential advantage of increased confidentiality, privacy and convenience when compared to testing undertaken by a health professional, thus reducing key barriers for some individuals.

Evidence from outside Europe suggests that HIVST is acceptable to MSM both in high and low-income settings globally [10, 11, 12, 13, 14]. Data suggests that MSM appreciate the confidentiality and privacy afforded by HIVST but some feel the lack of counselling services as a routine part of the testing process is problematic [11]; the ease of use has also been raised as a potential issue [12, 15]. While very few studies have evaluated post-test linkage with counselling and support or with treatment outcomes [10] there is little evidence that HIVST leads to unintended harm [14] nor any other significant unintended outcomes [12].

While evidence emerges about the acceptability and likely feasibility of delivering HIVST interventions to key populations (see [14] for the most recent review), none arises from England and very little from the rest of Europe [14]. Evidence to inform the design of an HIVST intervention for MSM in the UK is lacking. There is also a lack of evidence exploring how changes in the configuration of intervention components including delivery mechanisms and supportive strategies impact the acceptability of HIVST generally, an issue of particular relevance given that free HIV testing is readily available through a diverse array of other services.

This study aims to understand values and preferences for HIVST interventions targeting MSM in the UK. We explore the acceptability of HIVST among MSM in the context of known barriers and facilitators to testing for HIV and assess preferences for, and the concerns about, HIVST.

Our approach is embedded within implementation science, a field which seeks to translate and implement research evidence into policy and practice [16]. As such our results will be of particular interest to those seeking to understand the potential role of HIVST for MSM in the UK and other high resource settings (European and otherwise) with similar service provision (that is, good coverage of sexual health services for little or no cost).

## Methods

### Study design

This qualitative study sought to capture the perspectives of MSM in relation to HIV testing generally and HIVST specifically. Focus group discussions (FGDs) were selected in order to situate the perspectives of individual MSM in the context of group mediated normative understandings of HIV testing, such as those held within individuals’ social networks.

### Study sites and health service features

Fieldwork occurred in London, Plymouth and Manchester. These cities were chosen as they have a variable prevalence of HIV and differ in their population density of MSM. They also vary substantially in the provision and diversity of gay venues and HIV and STI testing services.

London (population 8.5 million) is exceptionally well served by specialist GUM clinics and has a range of community based testing initiatives run by both the statutory and voluntary sectors [17]. Manchester (population 511 000) has less extensively developed services compared to London, although there is good coverage with health service and voluntary sector HIV and STI testing available [17]. Plymouth (population 235,000) is a relatively small city and a regional centre, and in contrast has markedly less choice in sexual health care with one main GUM clinic and some provision from a voluntary sector organisation, both of which draw service users from across the counties of Cornwall and Devon [17]. At the time of the research, Plymouth was the only location in England piloting free NHS-provided HIVST. This was a time limited service in which 1000 tests were available for distribution.

### Study participants & recruitment

Gay, bisexual and other men who have sex with men (MSM) including trans men who were over the age of 18 and did not have diagnosed HIV were eligible for inclusion in this study.

Acknowledging differing patterns of testing across sub-groups, purposive quota sampling was used in order to ensure diversity regarding age, ethnicity, sexual orientation and past HIV testing experience including locations of previous HIV tests [2, 5]. In particular we emphasised including more men outside the ages of 26–39 years, as these men are less likely to test frequently [2, 4]. Further, we over-sampled ethnic minority men theorising that their barriers and motivators to testing may be different to men of White ethnicity. In our sampling strategy we particularly focused on including larger numbers of participants who had utilised self-administered testing or sampling methods including HIVSS and HIVST.

Sampling proceeded iteratively, and as study recruitment unfolded, we made efforts to recruit those who had never tested and men at potentially higher risk of HIV transmission on the premise that they might have greater need for HIVST given the UK testing guidelines recommending quarterly testing for these groups [18], and the potential for HIVST to provide a gateway to testing for men who have never tested [11]. The first four focus groups (two in London, one in Plymouth and one in Manchester) were shaped by our purposive sampling, with one additional group conducted with men reporting at least two male partners with whom they had condomless anal intercourse in the preceding three months, and one final group of men who had never previously tested for HIV.

Participants were recruited through gay location based social networking applications (Scruff, Growlr and Grindr) as well as community based organisations in the three cities. Men interested in the research were directed to a web-pages detailing information on the study and collecting consent to be contacted. They then filled in a short survey providing demographic details (presented in Table 1) and, if eligible, their contact details. Participants were then selected and invited to participate in groups based on our sampling frame. In all, one hundred and ninety-six individuals completed our screening survey, forty-seven of whom were invited to and subsequently attended an FGD. Participants were compensated £40.

**Table 1:** Participant demographic details.

Demographic features		MSM recruited
**Age group**	18–25 years	9
	26–39 years	21
	40+	17
**Ethnicity**	Asian	6
	Black	4
	White	37
	Mixed / other	0
**Sexual orientation**	Gay	38
	Bisexual	5
	Other (not gay or bi)	4
**Recency of HIV testing**	In last 12 months	30
	12+ months ago	9
	Never	8
**Past HIV testing locations**	GUM	30
**(multiple allowed)**	GP	6
	Community / PoCT	6
	Self-sampling	11
	Self-testing	4

### Data collection and analysis

FGDs were co-facilitated by the lead author and various members of Sigma Research, a research group at the London School of Hygiene and Tropical Medicine which focuses on the social, behavioural and policy aspects of HIV and sexual health. A topic guide was developed collaboratively within the research team and refined after the initial focus group. The topic guide was theoretically underpinned by the COM-B model of behaviour change which highlights how capability, opportunity and motivation impact on and interact with behaviour [19]. Our guide covered all three domains, including HIVST intervention specific details (opportunity and capability) and perceptions of HIVST in relation to other testing opportunities (motivation). During the section on intervention specific details, participants were asked to construct their ideal HIVST intervention choosing preferred options for sample type (blood vs saliva), window period (the time it takes for a test to detect an infection; 12 vs 4 weeks representing 2^nd^ and 4^th^ generation tests respectively), mode of instruction (written vs video), and access option (postal delivery or pick-up). Participants were handed cards with all options printed on separate pieces of paper, and asked to mark their preferred option between each pair. They then ranked importance of each domain (sample, window period, delivery and instructions) from 1 to 4.

OraQuick^TM^ saliva-based and BioSure^TM^ blood-based testing second generation HIV self-test kits were also demonstrated for participants who were asked to comment on procedure, design and instructions. The sensitivity and specificity of the tests was only commented on by facilitators if participants queried them.

FGDs were transcribed verbatim. All authors familiarised themselves with the transcripts and agreed a thematic coding framework through consensus. This framework took higher level codes such as barriers / facilitators, intervention preferences, and impacts; nested sub-themes described the most common understandings expressed by our participants. The data was initially coded at the higher level themes, then at sub-themes. Finally these sub-themes were coded iteratively where appropriate to derive more nuanced understandings of values and preferences. This analysis was conducted using QRS NVivo 10.

### Ethical considerations

Ethical approval for the MSM focus groups was sought from, and granted by, the ethics board at London School of Hygiene & Tropical Medicine (reference 9893).

## Results

Between July and November 2015, forty-seven MSM were recruited and attended a focus group. The sample was diverse, with a mean age of 36.1 years (range 20–64), more than 20% coming from Black and minority ethnic communities, 20% being not gay identified MSM and more than one third not following current UK HIV testing guidelines of annual tests for MSM [18]. We only asked men about condomless anal intercourse when recruiting to the higher-risk group so those data are unavailable for the majority of our sample, but in that group 9 men reported 2 or more anal sex partners with whom a condom was not used in the preceding 3 months. Location for past HIV tests was similarly varied, and more than 30% of our participants had accessed HIVSS or HIVST (see Table 1 for full demographic details).

Overall very few were outwardly opposed to HIVST with most describing it as highly acceptable, both as a general concept, and after specific discussion of the two test kits. We describe two key perceived benefits of HIVST- confidentiality and convenience–and two key potential drawbacks–concerns about the process and fear of the potential for a positive diagnosis with no immediate support. We also examine the key features of an ideal HIVST intervention and describe the perceived potential impact of widespread HIVST availability.

### Perceived benefits of HIVST

The primary perceived benefit was that HIVST (and to a lesser extent HIVSS) was assumed to be exceptionally useful for individuals who were concerned about privacy and confidentiality when accessing testing face-to-face. HIVST was widely assumed to afford a level of privacy that made HIV testing more accessible to people who otherwise found it difficult.

*I'm from up north and everybody knows everybody else*. *So people will see you go into that building* [GUM clinic], *and people will talk*. *Nothing is secret*, *but having the opportunity to have that sent away or getting it instantly over the counter eradicates all that embarrassment* (28-year-old gay man, London).

Except among men who had never tested, the importance of privacy was usually articulated on behalf of ‘other’ populations of MSM, and not for the speaker themselves. HIVST was perceived to be potentially beneficial for those that were not yet “out” about their sexuality, such as relatively young men, those who also had relationships with women, men living in rural areas, and those from ethnic and cultural communities where disclosure of homosexual activity remained taboo.

*My ex-partner was a Muslim and within his family*, *and all that*, *being gay is not allowed*. *But I think having a self-testing kit when he can do it at home in our home*, *I know he would appreciate that*. *I know he couldn’t take it back to where he's from*, *but in my home he can*. (51-year-old gay man, Manchester)

British Asian participants in particular identified HIVST as far preferable to accessing testing from GPs who were seen to have close links with family and community. The added privacy and confidentiality conferred by self-testing was also thought to be particularly important for individuals who lived outside major metropolitan areas or where there were concerns about being seen to use GUM clinics or asking for testing in primary care services.

HIVST was widely understood to be a technology used within the home. The confidentiality of the intervention was therefore somewhat undermined for participants who lived with family or other individuals with whom they were not open about their sexual activity.

The next most frequently cited benefit of HIVST was convenience, including the speed with which a test could be done and result obtained. The opportunity to test, when they had time, and wherever they were, was highly valued. This was true for individuals who lived in all areas of the country, but especially for those that struggled to access acceptable services because of long travel times, part-time clinic opening times, or appointments procedures.

*Well for me it’s an hour to drive here*, *and hour to go to Truro*. *Newquay is an option*, *and then that’s only certain mornings of the week and then it’s taking time off work to go*, *so it does get quite tricky*. (41-year-old gay man, Plymouth)

### Perceived drawbacks of HIVST

When considering HIVST, some participants had serious concerns about their capacity to perform a self-test. A few were averse to any possibility of self-administering a blood-based test and would only use a saliva-based HIVST, however more feared the process of self-testing, including the potential for errors in generating and interpreting the test outcome.

*I am quite clumsy and I am not good with instructions and I do not like to be told what to do*. *So*, *I think*, *how can you trust that it you have done it right? How can you trust that you can interpret the results correctly?* (25-year-old gay man, Manchester)

For some this performance anxiety was generalised–they simply had no experience of using a lancet or collecting a sample or interpreting a result, while for others the perceived volume and complexity of the written instructions was a major obstacle. Some specifically raised concerns about the high literacy level assumed by the instructions.

By far the most common cited barrier to using HIVST was the fear of having a reactive / positive result without any immediate personal support. These views were common across all groups but participants in the higher risk and never tested groups tended to express them more strongly.

*… if you do self-test and the results are positive*, *there’s the trauma as well of that*, *that person being by themselves having tested themselves and found out they’re positive* (62-year-old gay man, never tested group, London).

For some participants this could be mitigated by self-testing with a partner or friend/s, but for others having professional support available was crucial if there was any possibility of a positive/ reactive result. These men would either never use an HIVST or would only do so if they felt there was no chance of a positive result.

### Intervention preferences

In the intervention preferences exercise, the window period and sample type emerged as the two most important elements, with access options and instructions typically being seen as of lesser importance (Table 2). Below we explore expressed preferences for self-test attributes. These are mapped onto the COM-B domains they impact (alongside other HIVST attributes) in Table 3. We also describe whether each element is a barrier, facilitator, or could be either depending on the individual.

**Table 2:** Intervention preferences exercise results.

	Number of respondents ranking in position			
Test attribute	1	2	3	4
**Window period**	17	12	5	2
**Sample type**	12	13	9	3
**Access options**	7	5	13	12
**Instructions**	2	7	9	19

**Table 3:** HIVST attributes, components and relationship to COM-B domains.

**HIVST attributes**	**COM-B domains**	**Barrier / facilitator**
Choice	Opportunity	F
Confidentiality	Motivation, capability	E
Convenience	Motivation	F
Dislocation from care	Motivation	B
**Intervention specific components**	**COM-B domains**	**Barrier / facilitator**
Access options	Opportunity	F
Instructions	Capability	E
Sample type	Capability, motivation	E
Support	Motivation	B
Testing process	Capability	B
Window period	Motivation	E

Legend: B = Barrier; F = Facilitator; E = Either

In all our FGDs the window period was the most important element of a potential HIVST intervention, with 4^th^ generation testing commonly understood to be the gold standard. There was a strong feeling that for HIVST to have widespread utility, it would require a similar window period to a clinic test.

*I guess you don’t want it to be “oh crap*, *things went a bit crazy last week and I'll get this now and do it and oh this is a negative” and find that actually it’s much more like twelve weeks… I could imagine* [using HIVST] *but only if I could get a test* [where the] *window period was as good as a clinic test*. (20-year-old queer man, London).

Indeed, for many the perceived benefits of HIVST (privacy, convenience, immediacy) were eroded by the fact that all available self-tests at the time of the research were 2^nd^ generation when 4^th^ generation tests were available for no cost in other settings, including self-sampling services.

Blood based sampling was believed to be more accurate than saliva and there was a preference for these samples. The exception to this was the minority of MSM who had aversion to blood or needles who stated they could not utilise HIVST unless a saliva option was available despite the perceived accuracy limitations.

Postal delivery of kits was preferred over accessing through retail or healthcare settings as this option was seen to be exceptionally convenient. However, there was a high degree of concern that neither the BioSure^TM^ nor OraQuick^TM^ kits available at the time of the FGD would fit through standard letter boxes, potentially causing delivery problems. For an intervention to have widespread appeal, multiple access options were considered necessary.

Participants valued a range of mediums for instructions, with a slight preference for video. The test instructions were felt to make both tests seem significantly more complex to perform than they actually were. The nature of the packaging also led participants to be suspicious of the quality of the tests themselves because they were perceived to be over-produced and over-packaged.

The most favoured method of support was a telephone helpline ideally available 24 hours per day for individuals who test positive using HIVST and for those with a negative result who required additional support, particularly around risk reduction. This was seen as crucial to mitigate against the perceived potential for self-harm.

In the UK context, where HIV testing is free at a range of venues many participants reported being unwilling to pay for HIVST. Those who were willing to pay typically stated that they would pay the equivalent of travel costs to a clinic, plus a small amount of additional money for the convenience. This figure ranged from a low of £4 in London to £10 in Plymouth, probably reflecting the difference in accessibility of HIV testing in these cities.

### Potential impact of HIVST

A wide range of potential impacts of HIVST were discussed. While increased frequency of testing was often cited as a benefit of HIVST, there were significant concerns about the impact of dislocating HIV testing from STI counselling and testing services and STI/HIV care pathways. We explore these below.

It was assumed that among those who would use HIVST, the intervention would facilitate more frequent testing. The highly convenient nature of the intervention, particularly in relation to potential long wait or travel times to clinics, as well as the reduced potential for embarrassment meant that providing HIVST was assumed to have the potential to dramatically increase the proportion of gay men that test every year, and the frequency with which they do so.

*I don't think it’s only the inconvenience*, *I take my health care quite seriously and don't want to have HIV and I'm not cavalier about it*. *On the other hand I don't think I've been tested for about two years where as if I could pick one up of supermarket shelf I'd probably have done that test about ten times*. (38-year-old gay man, London)

However, participants tended to value time in GUM clinics, where staff were highly regarded for their role in educating and supporting patients about sexual risk management. While most pronounced in the higher risk group, concern existed for many that if individuals primarily tested through HIVST, this could lead to a de-skilling of themselves. This could be partially counteracted by providing enhanced sexual health information alongside HIVST, perhaps through a helpline or online.

*I learn so much from when I go to get tested*, *there’s always something new coming out or a trial or some sexual health information that I maybe didn’t know and if someone’s just carrying on their own doing it themselves*…(26-year-old gay man, higher risk group, London).

A concern for several participants was the potential for an increase in STIs. These concerns typically came under one of two themes. The first was that widespread use of HIVST, if provided without other testing for bacterial STIs, could lead to increases in infections as individuals would not be accessing full screening. The second, less common but related theme, was that people using HIVST might test with sexual partners as a strategy to avoid use of condoms. The concern was that this could lead to an increase in either STIs, or that individuals with acute HIV infection might unwittingly transmit HIV to a sexual partner because of the window period.

People self-harming following a reactive result was the most common concern raised. This fear was projected onto ‘others’, with individuals rarely identifying themselves or anyone in their immediate social groups as being at risk of self-harm.

Some also raised concerns that HIVST could potentially lead to people not linking into HIV clinical care services following an HIV positive result, and the impact that this could have on disease progression for the individual as well as implications for onward transmission. This concern was also exclusively related to ‘other’ men, and not the speaker themselves or to individuals in their social networks.

## Discussion

In our study of six FGD with 47 MSM in three UK cities, we found that HIVST was highly acceptable. MSM cited convenience and confidentiality as key benefits of the technology. Concerns about the testing process and in particular about the potential for a positive diagnosis using HIVST were commonly cited drawbacks. This is congruent with previous studies among MSM conducted largely in Australia, the USA and China (as well as emerging evidence from Scotland) indicating that HIVST is acceptable to MSM in a large part because of the associated privacy and ease of access, with concerns relating to support and capability in performing the tests [12, 14, 20].

### Intervention potential

An important finding of this study is the degree to which the intervention components (window period, sample type, access options, instructions and support) impact upon the acceptability of HIVST to those who might find it useful.

Fourth generation testing was of very high importance. This is particularly true as, in relation to all other testing methods, HIVST is felt to facilitate immediate knowledge of one’s HIV status, something undermined by a three-month window period. This poses potential questions as to the role of HIVST until 4^th^ generation tests are available given the availability of 4^th^ generation self-administered HIV testing methods through HIVSS services.

In contrast to much of the published literature [12, 14], we found a greater preference for blood based tests than for saliva sampling largely because participants felt that blood was a more accurate sample type. It is crucial to note however that for HIVST to have a wide appeal, a saliva option is also required otherwise those averse to needles or taking their own blood will be excluded.

It was clear that a range of access options were vital to ensure that the intervention was both confidential and convenient for a wide range of people with a diverse set of needs. The packaging and instructions of HIVST were also of importance to our participants, particularly given the high level of concern surrounding individuals’ own capabilities to perform HIVST. The instructions were seen to over complicate the testing process and led to a high degree of confusion and anxiety. The format and intricacy of the instructions were in the main developed in order to gain CE marking (crucial for certain products for sale in the European Economic Area) indicating that this issue will likely persist.

Through understanding HIVST attributes and their relationship to COM-B domains, promotion of HIVST can capitalise on facilitators by ensuring that intervention components support men’s values. Convenience and confidentiality in particular can be maintained by offering multiple access options while intelligent service design can make efforts to counteract significant barriers. Understanding and reducing barriers such as concerns around capability can be done through providing a range of instructions which should also enable motivational approaches to work more effectively.

Indeed, perhaps the greatest benefit of an HIVST intervention to commissioners and policymakers is the opportunity provided by the potential flexibility of HIVSTs; they should be able to cater to a range of needs within a population through provision of different kit options regarding sample type, access points and instructions. By understanding how elements in HIVST interventions impact on individual’s capability, opportunity, motivation and ultimately behaviour, service delivery can be tailored to suit the needs of particular groups, perhaps expanding testing to new sub-groups of MSM.

### Context of implementation

HIVST was thought likely to provide opportunity and increase motivation for more frequent testing among MSM given its convenience, confidentiality and accessibility. Given the low level of willingness to pay, this is particularly true should HIVST be widely available at no cost. This is encouraging for policy makers and health practitioners who aspire to lessen the interval between tests for all MSM [2], and the time between infection and diagnosis for those acquiring HIV [21].

The opportunity to test away from clinical settings was problematic for some participants who feared the dislocation of HIV testing from STI screening and current care pathways. This underlines the central role that sexual health services play in the sexual health of MSM. The anxiety surrounding HIVST is heightened by the increasing focus on self-care and diagnosis on a remote basis with sexual health interventions increasingly being delivered in the community and remotely. These shifts are driven partly by public health policies aiming to increase the variety of testing options available [2, 22], by cuts to public health budgets [22] and as part of broader shifts in how care and responsibility for care is governed [23].

Consistent with other published research [24] we found that MSM were unlikely to utilise HIVST as their primary testing method. This was because of concern about the dislocation of testing from other services and preference for 4^th^ generation HIV testing. Therefore, when designing interventions, HIVST should be considered a supplementary option which can increase the ability of individuals to test frequently while potentially diverting lower risk individuals from clinical services. For HIVST to be widely adopted, innovative strategies to embed HIVST within existing care pathways must be developed. In particular, it may be highly acceptable for HIVST to be delivered as part of an integrated package of care, include the provision of self-sampling kits for bacterial STIs and access to health advisor support if required. Using existing clinical services to manage the distribution of kits and provide care pathways may harness the widespread trust in GUM services and lend increased legitimacy to self-testing.

### Strengths and limitations

This manuscript presents the results of a formative qualitative study investigating values and preferences of a potential HIVST intervention among MSM in the UK. While HIV testing preferences and behaviours within this group have been extensively studied and documented, this is the first UK research describing preferences for HIVST interventions. This data will be exceptionally useful when considered alongside emerging evidence from Scotland which reports HIVST is highly acceptable among MSM and stakeholders [20].

Our results should be interpreted with some caution. For one, only 4 of our sample of 47 had previously used HIVST, so our results largely relate to perceptions of a new intervention. To counter this concern we over-sampled individuals who had accessed HIVSS, but there remain key differences between these interventions, particularly surrounding support and care pathways. Concerns around impacts will therefore potentially be over-emphasised and more research is needed to understand how these are borne out when HIVST is more widely used.

Further, as a study which is qualitative in nature, these findings should be understood as indicative of the diversity of values and preferences and their meanings among MSM in the UK, rather than representative of the entire population. We delineate how the context of implementation should shape the design and delivery of future HIVST service development and its evaluation.

## Abbreviations

HIVST: HIV self-testing
HIVSS: HIV self-sampling
MSM: Men who have sex with men
FGD(s): Focus group discussion(s)
